# Detection of Quantitative Trait Loci (QTLs) for Resistances to Small Brown Planthopper and Rice Stripe Virus in Rice Using Recombinant Inbred Lines

**DOI:** 10.3390/ijms14048406

**Published:** 2013-04-16

**Authors:** Qi Wang, Yuqiang Liu, Jinlong Hu, Yingxin Zhang, Kun Xie, Baoxiang Wang, Le Quang Tuyen, Zhaoqiang Song, Han Wu, Yanling Liu, Ling Jiang, Shijia Liu, Xianian Cheng, Chunming Wang, Huqu Zhai, Jianmin Wan

**Affiliations:** 1 State Key Laboratory for Crop Genetics and Germplasm Enhancement, Jiangsu Provincial Center of Plant Gene Engineering, Nanjing Agricultural University, Weigang 1, Nanjing 210095, China; E-Mails: 2009201052@njau.edu.cn (Q.W.); yql@njau.edu.cn (Y.L.); hjl114@163.com (J.H.); zyxrice@163.com (Y.Z.); 2009201008@njau.edu.cn (K.X.); wbxrice@163.com (B.W.); tuyensugarcane@yahoo.com.vn (L.Q.T.); zhaoqiangsong@sina.com (Z.S.); 2009201053@njau.edu.cn (H.W.); liuyanling198600@163.com (Y.L.); rice@njau.edu.cn (L.J.); liusj@njau.edu.cn (S.L.); xncheng@njau.edu.cn (X.C.); wangchm@njau.edu.cn (C.W.); zhaihq@mail.caas.net.cn (H.Z.); 2 Institute of Crop Science, the National Key Facility for Crop Gene Resources and Genetic Improvement, Chinese Academy of Agricultural Sciences, Beijing 100081, China

**Keywords:** RIL population, quantitative trait locus, *Oryza sativa* L

## Abstract

Small brown planthopper (SBPH) and rice stripe virus (RSV) disease transmitted by SBPH cause serious damage to rice (*Oryza sativa* L.) in China. In the present study, we screened 312 rice accessions for resistance to SBPH. The *indica* variety, N22, is highly resistant to SBPH. One hundred and eighty two recombinant inbred lines (RILs) derived from a cross of N22 and the highly susceptible variety, USSR5, were used for quantitative trait locus (QTL) analysis of resistances to SBPH and RSV. In a modified seedbox screening test, three QTLs for SBPH resistance, *qSBPH2*, *qSBPH3* and *qSBPH7.1*, were mapped on chromosomes 2, 3 and 7, a total explaining 35.1% of the phenotypic variance. *qSBPH7.2* and *qSBPH11.2*, conferring antibiosis against SBPH, were detected on chromosomes 7 and 11 and accounted for 20.7% of the total phenotypic variance. In addition, *qSBPH5* and *qSBPH7.3*, expressing antixenosis to SBPH, were detected on chromosomes 5 and 7, explaining 23.9% of the phenotypic variance. *qSBPH7.1*, *qSBPH7.2* and *qSBPH7.3*, located in the same region between RM234 and RM429 on chromosome 7, using three different phenotyping methods indicate that the locus or region plays a major role in conferring resistance to SBPH in N22. Moreover, three QTLs, *qSTV4*, *qSTV11.1* and *qSTV11.2*, for RSV resistance were detected on chromosomes 4 and 11. *qSTV11.1* and *qSTV11.2* are located in the same region between RM287 and RM209 on chromosome 11. Molecular markers spanning these QTLs should be useful in the development of varieties with resistance to SBPH and RSV.

## 1. Introduction

The small brown planthopper (SBPH), *Laodelphax striatellus* Fallén (Homoptera: Delphacidae), one of the most destructive pests in rice (*Oryza sativa* L.), is widely distributed in China and Southeast Asia. The SBPH sucks rice sap and causes yellowness, wilting and even death at the seedling and early spike formation stages. In recent years, damage caused by SBPH feeding has increased, and serious yield reductions have occurred as a consequence [1–3]. Rice viral diseases, such as rice stripe virus (RSV) and rice black-streaked dwarf virus (RBSDV), transmitted by SBPH, often cause major yield losses [4,5]. Rice stripe disease is one of the most serious rice diseases in China. Heavy infestations of SBPH can lead to yield reductions of 30%–40% and sometimes even complete losses in some fields in Jiangsu and Anhui provinces [6,7]. In recent years, RBSDV has become epidemic in Jiangsu and Zhejiang. This disease causes severe stunting and dark leaf symptoms on rice, maize, wheat and other cereal crops. Diseased plants often produce poor heads or none at all. Damage has been very severe, with disease incidence exceeding 90% in some areas, due to the widespread use of susceptible cultivars, abundance of the virus vector in fields and cultivation practices that favor infestation [8,9].

Rice stripe disease could be effectively controlled by reducing SBPH feeding. The disease has become more serious with higher incidences of the vector and severity of symptoms, which are directly related to the amount of virus transmitted by SBPH [10]. Similarly, rice black-streaked dwarf virus disease is also closely reflective of SBPH containing RBSDV [11,12]. Therefore, it is important to control SBPH, which currently depends mainly on pesticide applications, but with pesticide sprays, natural enemies are also killed, in addition to possible environmental pollution. With increases in chemical resistance and the migration behavior of SBPH, chemical control is not satisfactory [13–15]. Host resistance has been recognized as one of the most economic and effective measures in controlling SBPH, RSV and RBSDV. A few RSV resistance genes in rice have been reported [16–18]. Most of them show stable resistance and are located on the long arm of chromosome 11. However, varieties with single resistance genes are always at risk of their resistance being overcome by new strains of virus. There were no reported sources of high resistance to RBSDV, until recently [5,19]. It is therefore necessary to find additional sources of resistance to RSV and other viruses vectored by SBPH.

In order to search for novel SBPH and RSV resistance genes and to identify molecular markers linked to these genes, 312 rice accessions were screened for SBPH and RSV resistances; the *indica* cultivar, N22, showed strong SBPH and RSV resistances. A recombinant inbred line population of 182 lines was developed from the cross N22/USSR5 and was used to detect the quantitative trait locus (QTL) for resistance to SBPH and RSV.

## 2. Results

### 2.1. Screening Rice Varieties for Resistance to SBPH

**Figure 1:**
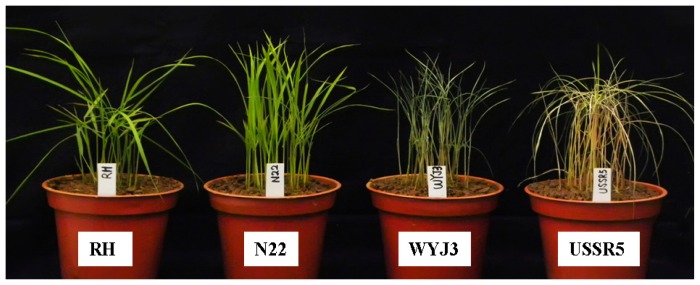
Phenotypes of parents and control varieties infested with SBPH in the modified seedbox screening test (MSST).

To screen rice varieties for resistance to SBPH, 312 accessions have been identified. The resistant control, Rathu Heenati (RH), showed no symptoms in the modified seedbox screening test (MSST), with a resistance rating of zero, whereas the susceptible control, Wuyujing3 (WYJ3), was rated 9.5. Similarly, RH showed significantly higher antixenosis and antibiosis than WYJ3. Among the 312 landraces and commercial varieties from different rice growing regions, 68 were highly resistant to SBPH, 25 were resistant, 93 were susceptible and 128 were highly susceptible (Table 1). Among the highly resistant varieties, 47.1% were *japonica* types and 52.9% were *indica*. Among the 128 highly susceptible lines, *japonica* and *indica* accounted for 85.2% and 14.8%, respectively. The Indian landrace, N22, was highly resistant, and the Japanese elite *japonica* variety, USSR5, was highly susceptible (Table 2 and Figure 1).

### 2.2. Construction of a Linkage Map with Simple Sequence Repeat (SSR) Markers

**Figure 2:**
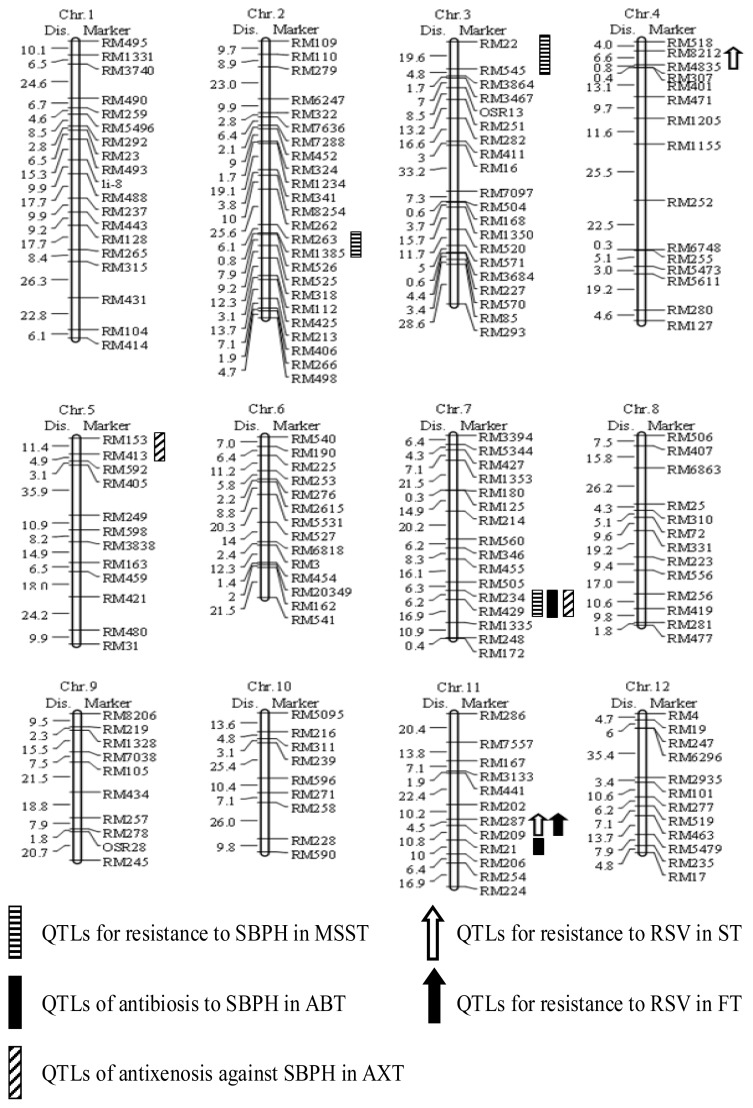
Molecular linkage map constructed by simple sequence repeat (SSR) markers assayed on the N22/USSR5 RIL population and quantitative trait loci (QTLs) conferring resistance to SBPH and rice stripe virus (RSV) using different methods.

To identify the locus for SBPH resistance, the recombinant inbred lines (RILs) population derived from a cross between the *indica* variety, N22, and the *japonica* variety, USSR5, was developed by single-seed descent. Three hundred and forty markers distributed across all chromosomes of the rice genome were polymorphic between N22 and USSR5. A molecular map with 176 simple sequence repeat (SSR) markers was constructed using data from the N22 × USSR5 RILs. The total map length was 1702.8 cM with an average distance between markers of 9.7 cM. The percentage of the USSR5 genome in each line ranged from 23.5% to 80.1% with an average of 49.4%, not significantly different from the expected 50%. The segregation ratios of the two genotypic classes for most loci fitted expected Mendelian ratios of 1 (N22):1 (USSR5) (Figure 2).

### 2.3. Evaluation of SBPH Reaction and QTL Analysis

**Figure 3:**
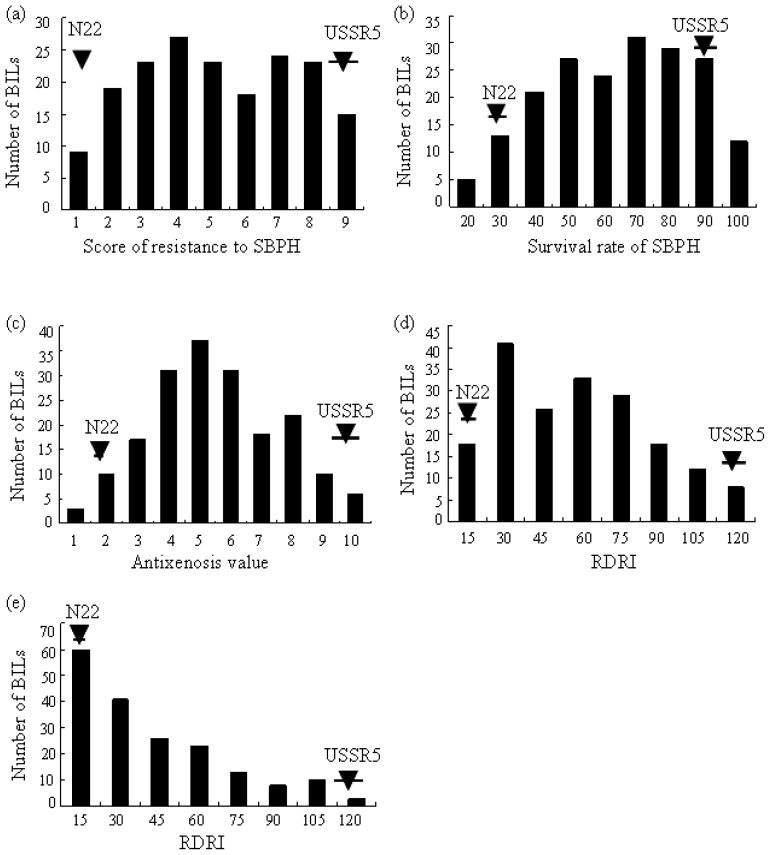
Distribution of SBPH and RSV reactions in the N22/USSR5 RIL population. (**a**) Modified seedbox screening test (MSST) for SBPH; (**b**) antibiosis test (ABT) for SBPH; (**c**) antixenosis test (AXT) for SBPH; (**d**) seedling test (ST) for RSV; (**e**) field test (FT) for RSV. Arrowheads indicate values of parental varieties. The error bars represent the standard derivation (*n* = 4).

The resistant score of N22 and USSR5 in MSST were 1.5 and 9.2, respectively. The response scores of the 182 N22/USSR5 RILs were continuously distributed over a range from 1.0 to 9.0 in MSST, indicating a polygenic control of the resistance to SBPH in this population (Table 2 and Figure 3). Three QTLs for SBPH resistance, designated *qSBPH2*, *qSBPH3* and *qSBPH7.1*, were mapped on chromosomes 2, 3 and 7 by composite interval mapping with LOD scores of 2.33, 2.54 and 3.42. These QTLs explained 10.0%, 7.7% and 17.4%, respectively, of the phenotypic variation in this population, (Figure 2). As indicated by additive effects, the resistance alleles, *qSBPH2*, *qSBPH3* and *qSBPH7.1*, were from USSR5, N22 and N22, respectively (Table 3).

### 2.4. Antibiosis Test and QTL Analysis

The survival rate of SBPH of parental varieties, N22 and USSR5, were 31% and 95%, respectively, indicating that N22 provided relatively strong antibiosis to SBPH. The continuous distribution of the survival rate of nymphs ranging from 21% to 100% showed that several genes governed antibiosis in the RIL population (Table 2 and Figure 3). Two QTLs, with logarithm of odds (LOD) scores of 3.30 and 2.60 and designated *qSBPH7.2* and *qSBPH11* conferred antibiosis to SBPH. These genes were mapped on chromosomes 7 and 11, accounting for 13.2% and 7.5%, respectively, of the phenotypic variance in the RIL population (Table 3 and Figure 2).

### 2.5. Antixenosis against SBPH and QTL Detection

Antixenosis is the ability of a variety to repel insects, causing a reduction in feeding or oviposition. Antixenosis value is generally measured by comparing the number of insects landing on different test varieties. The antixenosis values of N22 and USSR5 were 2.0 and 9.0, respectively, and were significantly different. This result showed N22 conferred strong antixenosis against SBPH. Continuous and transgressive segregation was also observed in the RIL population, with a range of insect numbers from 1.0 to 10.0. The normal distribution of antixenosis values indicated that minor genes controlled antixenosis to SBPH (Table 2 and Figure 3). Two QTLs, *qSBPH5* and *qSBPH7.3*, conferring SBPH antixenosis, were detected on chromosomes 5 and 7 in the regions RM153-RM413 and RM234-RM429 with LOD scores of 2.51 and 3.40, respectively. These QTLs explained 23.9% of the total phenotypic variance in the RIL population (Figure 2 and Table 3).

### 2.6. QTL Analysis of RSV Resistance in the RIL Population

The relative disease rating index (RDRI) of N22 and USSR5 were 8.3–10.3 and 115.2–120.8, respectively, when assessed by two infection methods, respectively, and showed that N22 was highly resistant to RSV, whereas USSR5 was susceptible. The RDRI of the RIL population showed a continuous distribution with transgressive segregation in both the field test (FT) and seedling test (ST) (Table 2 and Figure 3), indicating polygenic control of resistance. QTLs for RSV resistance were detected on chromosomes 4 and 11. The former was detected only in FT conditions, with a LOD score of 5.20, explaining 13.4% of the phenotypic variance. *qSTV11.1* and *qSTV11.2* on chromosome 11 were detected in FT and ST infected conditions, where it explained 30.2% and 28.9% of the phenotypic variance, with LOD scores of 8.58 and 8.03, respectively. The resistance effect at both loci came from N22 (Table 2).

## 3. Discussion

Despite low yield and poor agronomic traits, landraces often have high resistance to biotic stresses. Natural variation present in landraces has played a vital role in breeding for resistance to biotic and abiotic stresses. For example, RSV resistance gene, *Stvb-i*, originated from Pakistani landrace, Modan, and the brown planthopper (BPH) resistance gene, *bph2*, originated from ASD7. These genes were subsequently used in many commercial varieties. Here, we identified 13 highly resistant and 66 resistant accessions by screening landraces from different regions, thus providing additional germplasm sources for SBPH resistance breeding. In our tests, most *indica* types were highly resistant, whereas *japonica* accessions tended to be susceptible, consistent with previous studies [20]. Similarly, both BPH and whitebacked planthopper (WBPH) resistances were reported to be rare in *japonica* germplasm; however, both have been found in *indica* types and in certain wild relatives [21,22]. Thus, it will be worthwhile to screen for resistance in *indica* types, and such varieties will constitute valuable breeding materials for developing of rice varieties resistant to planthoppers, as well as being excellent differentials for basic studies on the nature of insect resistance in plants. The Indian landrace, N22, showed highly resistant to SBPH in the present screening for the SBPH resistance resource. In order to identify novel resistance genes for SBPH and RSV, the N22/USSR5 recombinant inbred lines (RILs) population was developed.

### 3.1. Genetic Mechanisms of Resistance to SBPH in “N22”

QTL analysis of different resistance phenotypes will reveal the genetic mechanisms of resistance and indicate those alleles conferring more stable resistances for use in germplasm improvement and breeding for resistance. Several QTLs for SBPH resistance were identified through three phenotypic systems using the N22/USSR5 recombinant inbred lines (RILs) population (Table 2 and Figure 2). The MSST phenotyping scale provides an accumulative measure of antixenosis, antibiosis and tolerance. Three QTLs for SBPH resistance were located on chromosomes 2, 3 and 7, accounting for 35.1% of the total phenotypic variance. In addition, two QTLs associated with antibiosis and two QTLs for antixenosis were identified, explaining 20.7% and 23.9% of the total phenotypic variances, respectively. The results suggested that both antibiosis and antixenosis contributed to protection against SBPH in N22.

Antixenosis and antibiosis tests reveal mechanisms of resistance, which are especially valuable in assessing *SBPH* reaction. Entries with antixenosis to viruliferous *SBPH* may markedly decrease planthopper feeding. For example, accessions releasing volatile repulsive chemicals may deter planthopper settlement and probing and, thus, greatly reduce the chance of RSV transmission. Furthermore, even if the entries with antixenosis do increase tentative probing, the chance of transmission of RSV may be further reduced in that successful transmission of RSV needs more than 30 min of continual feeding [23]. Genotypes with antibiosis can cause pests to have abnormal growth and development, thereby decreasing feeding; tolerance, on the other hand, usually does not affect insect feeding. Therefore, an understanding of the mechanisms of resistance should be useful in developing resistant varieties with high levels of antixenosis and/or antibiosis. The results of the present study indicate that several QTLs associated with tolerance, antibiosis and antixenosis jointly governed small brown planthopper resistance in N22.

### 3.2. A Reliable QTL for SBPH Resistance Detected on the Long Arm of Chromosome 7

Several QTLs against SBPH have been mapped using different populations (Table 4). Duan *et al*. used two different mapping populations to perform QTL analysis for resistance to SBPH, reporting two QTL in the region XNpb202-C1172 and S2260–G257 on the chromosome 11 derived from the varieties, DV85 and Kasalath, respectively [20,24]. These two QTL were repeatedly detected in MSST, antixenosis and antibiosis tests, indicated that these two QTL were important in conferring the resistance SBPH. On the other hand, Zhang *et al*., Duan *et al.* and Le *et al*. also detected two stable QTL on chromosome 11 and 12 using different mapping populations [25–27]. Comparing QTLs for SBPH resistance identified in this study with those already reported in the literature, it seems that the QTL against SBPH detected in this study are novel. QTLs identified in our study, *qSBPH7.1*, *qSBPH7.2* and *qSBPH7.3*, were all detected in the RM234-RM429 region on chromosome 7 using three phenotypic evaluation systems. The QTL *qWbph1* conferring resistance to whitebacked planthopper (*Sogatella furcifera* Horváth) (WBPH) derived from N22 and the QTLs conferring resistance to WBPH and brown planthopper (BPH) in IR64 were also mapped in the same region with *qSBPH7.1* (*qSBPH7.2* or *qSBPH7.3*) [28–31]. In addition, many studies have shown that the region near the RM234-RM429 interval on chromosome 7 harbors genes/QTLs for resistance to biotic and abiotic stresses. For example, major QTL for rice blast resistance were detected in the adjacent RM429 region of chromosome 7 in different populations [32–35]. Genes for resistance to abiotic stress, including cold stress [36], salt stress [37,38] and drought stress [39], have been identified around the RM234-RM429 interval. Based on the evidence discussed above, it is suggested that genes in this region harboring resistance to biotic and abiotic stresses will be useful for the development of varieties resistant to insects, diseases and/or abiotic stresses by marker-assisted selection. This observation indicated that the variety N22 is a desirable parent for pest resistance breeding. These QTLs harboring SBPH resistance in N22 are important and useful genes for pest resistance breeding.

### 3.3. The Inheritance of the RSV Resistance Present in “N22”

For RSV resistance, QTLs were detected on chromosomes 4 and 11. *qSTV4,* located between markers RM4835 and RM8212, accounted for 13.4% of the phenotypic variance. *qSTV11.1* and *qSTV11.2* were detected in the same region, RM287-RM209, on chromosome 11 L, in both field and seedling tests. In different varieties, many RSV resistance loci have been mapped in the same region as *qSTV11.1* or *qSTV11.2*; for example, *Stvb-i* in Modan [16], *qSTV11^KAS^* in the variety, Kasalath [17], and *qSTV11^TQ^* in Teqing [18]. The present *qSTV11.1* and *qSTV11.2* may be the same as one of these genes/QTLs, and this implicated that the QTL was expressed stably and independently in the genetic background. Resistance to RSV can be achieved either by resistance to the virus *per se* or by resistance to the SBPH vector. The former can be achieved either by inhibition of virus movement within the plant or suppression of its reproduction within plant cells through formation of necrotic spots. The latter can be achieved by introducing feeding tolerance, antixenosis or antibiosis. Based on our results, the RSV and SBPH resistance QTLs were located in different regions on chromosomes, indicating that resistance to RSV and SBPH are controlled by different QTLs in “N22”. Therefore, a combination of RSV and SBPH resistance QTLs/genes would be most desirable as a means of generating durable and stable resistance to rice stripe virus.

## 4. Experimental Section

### 4.1. Plant Materials

For SBPH resistance studies, the varieties, Rathu Heenati (*indica*) (RH) and Wuyujing #3 (*japonica*) (WYJ3), were used as resistant and susceptible controls. A total of 312 rice accessions (299 landraces and 15 commercial varieties from Japan, Korea, the International Rice Research Institute (IRRI) and China) were screened for reaction to SBPH (Table S1). *Indica* variety IR36 was used as the resistant control and WYJ3 as the susceptible control for RSV.

### 4.2. Insect Population

SBPH used for infestation were originally collected from a rice field at Nanjing and were maintained on rice plants in a greenhouse for four generations before being transferred to WYJ 3 plants in a greenhouse at the Rice Research Institute, Nanjing Agricultural University. The SBPH population for evaluating SBPH reaction was confirmed to be non-viruliferous by an immunobinding dot assay and RT-PCR detection. Similarly, for RSV reaction studies, the percentage of virus-containing SBPH was estimated to be 39% by random sampling and ELISA analysis [40,41].

### 4.3. Inoculation Methods

Three inoculation methods were used in tests for SBPH reaction. These were as follows.

(1) Modified seedbox screening test (MSST): a modified seed-box screening test was applied to evaluate reactions of 312 rice accessions and control varieties, as well as the parents and 182 BILs, as described previously [20]. To evaluate each genotype, about 60 uniformly germinated seeds of each line were sown in an 8 cm diameter plastic pot with a hole in the base. Generally, 28 pots, together with one pot of each parent and the control variety, were placed in a 65 × 44 × 14 cm plastic seedbox. All seedlings under evaluation were incubated at 26 ± 2 °C in sunlight. About 2 cm of water was maintained in the bottom of the seedbox. At the 1.5- to 2.0-leaf stage, seedlings were infested with second to third instar SBPH nymphs at 15 insects per seedling. Scoring of all materials in each seedbox according to the standard evaluation systems [42] was conducted when more than 90% of Wuyujing 3 seedlings were dead at 14 ± 1 days after infestation. The score for each entry was then calculated based on the weighted average of the number of seedlings tested (Table 5).
(2) Antixenosis test (AXT): following Duan *et al*. [20], 15 germinated seeds of each entry were grown in a row in a 65 × 44 × 14 cm plastic seedbox at 26 ± 2 °C. At the 1.5- to 2.0-leaf stage, seedlings were transferred into cages covered with nylon nets and infested with second to third instar SBPH nymphs at a rate of five insects per seedling. The number of insects was counted on each seedling at 8:00 and 16:00 daily, and the insects were then dispersed in order to distribute them evenly among seedlings after counting every day [43]. The average number of insects on each entry was calculated and regarded as the score value of antixenosis after 5 days.
(3) Antibiosis test (ABT): following Duan *et al*. [20], 5 germinated seeds for each entry (4 replicates) were grown in a 6 cm diameter × 15 cm high glass at 26 ± 2 °C. At the 1.5- to 2.0- leaf stage, seedlings were infested with 1–2 instar SBPH nymphs at a rate of 20 insects per glass. At 10 days after infestation, the survival percentage of insects on each variety was calculated and regarded as the antibiosis value.

For evaluation of responses to RSV, two inoculation methods were used. These were as follows.

(1) A field test (FT) done in a paddy field at Nanjing. Field trials were conducted in randomized complete blocks with two replicates. Sixty seeds of each RIL were sown in a 40 × 60 cm area on 10 May 2009. Weak seedlings were eliminated until ~40 seedlings remained at the 2.5 leaf stage. Wheat surrounding the paddy field was harvested on 5 June, and imagoes of SBPH were transferred to the rice seedlings. No pesticide was used during the entire growth period.
(2) A seedling test (ST) followed Sakurai *et al*. [44] with a few modifications: 30 germinated seeds of each line were sown in plastic dishes filled with soil. Weak seedlings were eliminated at the one leaf stage and 25 healthy seedlings of each line were kept for infestation. First to second instar SBPH nymphs were released into dishes covered with plastic cylinders at the rate of about five nymphs per seedling, when the seedlings were at the 1.5 leaf stage. During the infestation period*,* the insects in each dish were dispersed every day to avoid aggregation. Three days later, all SBPH nymphs were killed with pesticide, and seedlings were transferred to a greenhouse, where they produced symptoms after about one month. The experiments were performed with four replications. A relative disease rating index (RDRI = DRI × 100/the value of WYJ3) was calculated for each line, and QTL analysis was conducted, excluding the effect of the environment [45].

### 4.4. Genetic Linkage Map and QTL Analysis

Linkage groups and orders of markers were determined using MAPMAKER/EXP 3.0 [46]. The Kosambi mapping function was used to convert recombination frequencies to genetic distances (cM) [47]. QTL analysis of RILs was performed using a composite interval mapping method in Windows QTL Cartographer version 2.5 [48]. A permutation number of 1000 was applied for each trait in QTL analysis. The thresholds of LOD for all traits are the same: 2.5. The relative contribution was calculated as the percentage of phenotypic variation explained (PVE, %) by the QTL. The percentages of variation explained by a QTL and the additive effect were also estimated with the software. QTL were named according to McCouch *et al*. [49].

## 5. Conclusions

SBPH is an economically important pest in rice, not only causing direct damage by sucking plant sap, but also transmitting virus diseases, such as RSV and RBSDV, which often cause major yield losses. Host resistance has been recognized as one of the most economic and effective measures in controlling SBPH. In this study, 312 rice accessions were screened for their response to SBPH. An *indica* variety, N22, showed strong resistance to SBPH and RSV. Recombinant inbred lines (RILs) derived from a cross of N22 and the highly susceptible variety, USSR5, were used for QTL analysis of resistances to SBPH and RSV. Seven QTLs for SBPH resistance were detected on four different chromosomes. The QTL between RM234 and RM429 on chromosome 7 was detected repeatedly by all three phenotyping methods, indicating that the effect of this QTL is actual and stable. Additionally, two QTLs for resistance to RSV were also identified. Our study confirmed that the cultivar, N22, was highly resistant to SBPH, and *qSBPH7.1*, *qSBPH7.2* or *qSBPH7.3* should be an important locus for attention by breeders and researchers.

## Figures and Tables

**Table 1:** Small brown planthopper (SBPH) reactions of 312 rice landraces and varieties from different regions.

Origin Province/Country	Classification a												Total

	*Japonica* Type						*Indica* Type						
	
	I	HR	R	MR	S	HS	I	HR	R	MR	S	HS a	
Jilin						8							8
Heilongjiang			1		1	4							6
Liaoning				2	1	8							11
Shandong		1	2	2	2	2							9
Shanxi			1			1							2
Sichuan								1		2	1	3	7
Guizhou						1			1		1	1	4
Yunnan			1	2	5	4		1	1	5	2	6	27
Anhui					1	3			6	4	1	5	20
Jiangxi									2	1	8	4	15
Hubei						3					1	1	5
Hunan			1					3	5	4	1	1	15
Guangdong									3	2	3	7	15
Guangxi									2	2	5	3	12
Fujian									5	1	2	3	11
Zhejiang			2	1	3	4			2	1	1	1	15
Jiangsu			3	2		4		1	2				12
Taiwan			1	1	1	5					2	2	12
Taihu Valley			6	3	9	15			1			1	35
IRRI		4	7	6	2	1							20
India		1	2	1									4
South Korea				1	1	1			1	2			6
Malaysia			1	1	3	1							6
Indonesia			2	2		1							5
Other		1	1	2	4	11			4	2	3	2	30
Total		7	31	26	33	77		6	35	26	31	40	312

a I, immune; HR, highly resistant; R, resistant; MR, moderately resistance; S, susceptible; HS, highly susceptible.

**Table 2:** The phenotypic performance of the recombinant inbred lines (RILs) population and its parents with respect to SBPH infestation.

Test Method	Control *		Variety		RILs Population	
		
	WYJ3	RH	USSR5	N22	mean	range
Evaluation of SBPH resistance						
MSST	9.5 ± 0.8 a	0 c	9.2 ± 0.4 a	1.5 ± 0.2 b	5.2	1.0–9.0
ABT	98.0 ± 0.5 a	10.0 ± 0.7 c	95.0 ± 0.6 a	31.0 ± 1.3 b	60.1	21.0–100.0
AXT	9.2 ± 0.6 a	0.8 ± 0.2 c	9.0 ± 0.2 a	2.0 ± 0.3 b	5.8	1.0–10.0

* Rathu Heenati (RH) and Wuyujing3 (WYJ3) were the resistant and susceptible controls for SBPH infestation, respectively. Numbers followed by the different letters are significantly different at *p* < 0.05.

**Table 3:** QTLs for SBPH and RSV resistances detected in the N22/USSR5 RIL population.

Phenotyping Method	QTL	Marker Interval	Chromosome	LOD Score	PVE (%) a	Additive Effect b
Modified seedbox screening test	*qSBPH2*	RM263-RM1385	2	3.03	10.0	0.81
	*qSBPH3*	RM22-RM545	3	2.54	7.7	−0.72
	*qSBPH7.1*	RM234-RM429	7	3.42	17.4	−1.23

Antibiosis test	*qSBPH7.2*	RM234-RM429	7	3.30	13.2	−10.3
	*qSBPH11*	RM209-RM21	11	2.60	7.5	−5.4

Antixenosis test	*qSBPH5*	RM153-RM413	5	2.51	8.2	−0.37
	*qSBPH7.3*	RM234-RM429	7	3.40	15.7	−9.36

Seedling test	*qSTV4*	RM8212-RM4835	4	5.20	13.4	−3.19
	*qSTV11.1*	RM287-RM209	11	8.58	28.9	−7.77

Field test	*qSTV11.2*	RM287-RM209	11	8.03	30.2	−8.90

a , Percentage of phenotypic variation explained;

b , additive effect of QTLs detected in RIL population. Negative values indicate resistance alleles are contributed by “N22”; positive values indicate resistance alleles from “USSR5”. LOD logarithm of odds; PVE, phenotypic variation explained.

**Table 4:** QTLs for SBPH resistance reported up to 2013.

Chromosome	QTL	Linked Marker	Population	Reference
1	*qSBPH1*	C949–GA506	ZYQ8/JX17 DH a lines	Zhang *et al*. [25]

2	*qSBPH2*	RG322–CT41	ZYQ8/JX17 DH lines	Zhang *et al*. [25]
	*Qsbph2*	R1843–R712	Nipponbare/Kasalath//Nipponbare BILs b	Duan *et al*. [24]
	*Qsbph2b*	RM5791-RM29	Mudgo/Wuyujing 3 F_2:3_ lines	Duan *et al*. [26]

3	*Qsbph3b*	XNpb74-XNpb144	Kinmaze/DV85 RILs	Duan *et al*. [20]
	*Qsbph3b*	C80-C1677	Nipponbare/Kasalath//Nipponbare BILs	Duan *et al*. [24]
	*Qsbph3c*	R2170–C1135	Nipponbare/Kasalath//Nipponbare BILs	Duan *et al*. [24]
	*Qsbph3d*	RM3199–RM5442	Mudgo/Wuyujing 3 F_2:3_ lines	Duan *et al*. [26]

4	*qSBPH4*	RM451–RM5473	02428/Rathu Heenati F_2_ population	Le *et al*. [27]

8	*Qsbph8*	C390-R1943	Nipponbare/Kasalath//Nipponbare BILs	Duan *et al*. [24]

11	*Qsbph11a*	R2918-C410	Kinmaze/DV85 RILs c	Duan *et al*. [20]
	*Qsbph11b*	XNpb202-C1172	Kinmaze/DV85 RILs	Duan *et al*. [20]
	*Qsbph11c*	XNpb202-C1172	Kinmaze/DV85 RILs	Duan *et al*. [20]
	*Qsbph11d*	XNpb202-C1172	Kinmaze/DV85 RILs	Duan *et al*. [20]
	*Qsbph11d*	R1506–C950	Nipponbare/Kasalath//Nipponbare BILs	Duan *et al*. [24]
	*Qsbph11e*	S2260–G257	Nipponbare/Kasalath//Nipponbare BILs	Duan *et al*. [24]
	*Qsbph11f*	S2260–G257	Nipponbare/Kasalath//Nipponbare BILs	Duan *et al*. [24]
	*Qsbph11g*	S2260–G257	Nipponbare/Kasalath//Nipponbare BILs	Duan *et al*. [24]
	*qSBPH11h*	RG211–PTA818	ZYQ8/JX17 DH lines	Zhang *et al*. [25]

12	*Qsbph12a*	I12-17–RM3331	Mudgo/Wuyujing 3 F_2:3_ lines	Duan *et al*. [26]
	*qSBPH12*	RM519–RM3331	02428/Rathu Heenati F_2_ population	Le *et al*. [27]

a Doubled-haploid lines;

b backcross inbred lines;

c recombinant inbred lines.

**Table 5:** Evaluation criteria for seedling reaction to SBPH.

Symptoms	Score	Reaction a
No visible damage	0	I
Very slightly damage	1	HR
Partial yellowing of the first and the second leaves	3	R
Pronounced yellowing and some seedlings slight stunting	5	MR
Seedlings showing signs of wilting and severe stunting	7	S
Seedlings dead	9	HS

a I, immune; HR, highly resistant; R, resistant; MR, moderately resistance; S, susceptible; HS, highly susceptible.
